# Dietary Inflammatory Index and Blood Inflammatory Markers in Young Men with Different Levels of Physical Activity: A Cross-Sectional Observational Study

**DOI:** 10.3390/ijms27093994

**Published:** 2026-04-29

**Authors:** Anna Pietrzak, Anna Kęska, Michalina Błażkiewicz, Szymon Kuliś

**Affiliations:** 1 Research and Development Department, Ekamedica Ltd., Bielska 78a, 43-340 Kozy, Poland; anna.pietrzak@awf.edu.pl; 2 Faculty of Physical Education, The Józef Piłsudski University of Physical Education in Warsaw, 00-968 Warsaw, Poland; 3 Faculty of Rehabilitation, The Józef Piłsudski University of Physical Education in Warsaw, 00-968 Warsaw, Poland; michalina.blazkiewicz@awf.edu.pl

**Keywords:** Dietary Inflammatory Index (DII), inflammation, physical activity, diet assessment, serum amyloid A

## Abstract

Systemic inflammation is influenced by regular physical activity and diet. While moderate exercise can transiently alter inflammatory markers, high-intensity activity may increase muscle turnover without substantially elevating systemic inflammation. The combined effects of physical activity and dietary inflammatory potential in healthy young men remain poorly defined. In this cross-sectional observational study, 233 healthy men aged 18–30 years were categorized according to physical activity level: low (NA, *n* = 52), moderate (A, *n* = 93), and high (S, *n* = 88). Anthropometry and body composition were assessed using bioelectrical impedance. Dietary intake was recorded over 4 days and used to calculate the Dietary Inflammatory Index (DII). Blood samples were collected and analyzed for complete blood counts, high-sensitivity C-reactive protein (hs-CRP), serum amyloid A (SAA), and creatine kinase (CK). Differences between groups were evaluated using the Kruskal–Wallis test with Dunn’s post hoc correction, and principal component analysis (PCA) was performed to explore multivariate inflammatory patterns. The highest BMI, fat percentage, and DII were observed in low-activity men, whereas fat-free mass and CK activity were greatest in highly active men. Slightly higher systemic inflammatory markers (hs-CRP and SAA) were observed in moderately active men compared to other groups. PCA revealed two principal axes: PC1 representing systemic inflammation and PC2 representing leukocyte distribution. Weak associations were found between DII and these components, indicating a limited link between dietary inflammatory potential and circulating inflammatory biomarkers. Body composition is strongly influenced by physical activity, with high activity promoting lean mass and moderate activity associated with modest elevations in inflammatory markers. Dietary inflammatory potential was only weakly associated with systemic inflammation, suggesting that exercise-induced physiological stress may play a more prominent role in shaping inflammatory profiles in healthy young men.

## 1. Introduction

Regular physical activity is widely recognized as a cornerstone of health, contributing to optimal body composition, metabolic function, and immune regulation. Moderate exercise is generally associated with reduced systemic inflammation and lower risk of chronic diseases, whereas insufficient or extreme levels of physical activity can alter inflammatory and hematological parameters [1]. High-intensity or prolonged exercise can transiently elevate markers such as C-reactive protein (CRP) and serum amyloid A (SAA), reflecting physiological responses to muscle stress and repair [2].

Diet also plays a pivotal role in modulating systemic inflammation. The Dietary Inflammatory Index (DII) was developed as a literature-based tool to assess the inflammatory potential of an individual’s diet, with higher scores indicating pro-inflammatory patterns and lower scores reflecting anti-inflammatory intake. Elevated DII scores have been linked to increased concentrations of CRP, interleukin-6 (IL-6), and other inflammatory biomarkers across diverse populations, highlighting the impact of dietary patterns on immune function [3,4,5]. Conversely, anti-inflammatory diets are associated with reduced systemic inflammation and improved metabolic outcomes.

Although the individual effects of physical activity and diet on inflammation are well-established, their combined influence, particularly in healthy young men, remains poorly understood. Young adults represent a population with substantial variability in physical activity levels, ranging from sedentary behavior to intensive athletic training, which may differentially affect systemic inflammatory markers, leukocyte distribution, and body composition [6,7]. Additionally, early adulthood is a period when dietary habits often change, particularly among students, as confirmed by studies on weight gain among young adults, especially men, a phenomenon known as ‘Freshmen 15’ [8,9]. An increase in body fat promotes the development of chronic inflammation, which may, in the future, increase the risk of conditions such as cardiovascular disease, insulin resistance, and metabolic disorders. Assessing these associations is important for understanding lifestyle influences on early-life health trajectories and preventive strategies for long-term disease risk. This is especially important for physically active individuals, as a well-balanced diet supports recovery, accelerates the reduction in post-exercise inflammation, and enhances overall physical performance.

The primary aim of this study was to investigate the associations between physical activity level, dietary inflammatory potential, and systemic inflammatory markers in young adult men. Specifically, the study sought to:

1. Compare anthropometric, body composition, hematological, and biochemical characteristics among young men with low, moderate, and high physical activity levels.
2. Evaluate the dietary inflammatory potential using the Dietary Inflammatory Index (DII) and its relationship with inflammatory biomarkers.
3. Explore multivariate patterns of systemic inflammation and leukocyte distribution through principal component analysis, assessing whether these patterns differ according to physical activity level.

By integrating anthropometric, dietary, and inflammatory data, the study aimed to provide insights into how varying lifestyles influence inflammation and immune function in a healthy young male population.

## 2. Results

### 2.1. Anthropometric and Body Composition Profiles of the Study Participants

Of the 250 participants initially enrolled, 17 were excluded due to incomplete participation or missing data, resulting in a final analytical sample of 233 participants. No missing anthropometric, dietary, or laboratory data were present among the 233 participants included in the final analyses. The analysis of anthropometric and body composition characteristics revealed significant differences across the three study groups (NA: low, A: moderate, S: high) (Table 1). Age differed notably among the groups, with the moderate group being younger than both the low and high groups. Body mass showed a similar pattern, where participants in the moderate group weighed less than those in the low and high groups, while body height did not differ significantly across groups.

BMI values were lowest in the moderate group and higher in both the low and high groups, reflecting differences in overall body composition. Basal metabolic rate (BMR) was highest in the high group and significantly greater than in both the moderate and low groups, indicating a higher energy expenditure in individuals with higher body composition measures.

Measures of adiposity also varied markedly. The low group exhibited substantially higher fat percentage and fat mass compared to the moderate and high groups, while fat-free mass (FFM) was greatest in the high group and lowest in the low group, suggesting a shift from fat to lean tissue across the groups. Indicators of central adiposity, including waist-to-height ratio (WHtR) and waist-to-hip ratio (WHR), were elevated in the low group compared to the other groups, with all pairwise comparisons showing significant differences.

Overall, these results highlight that the moderate group had the lowest overall fat accumulation and BMI, while the high group had the greatest fat-free mass and basal metabolic rate. The low group, in contrast, displayed the highest adiposity and central fat distribution, reflecting a clear distinction in body composition profiles among the study groups.

### 2.2. Hematological and Biochemical Parameters Across Groups A, NA, and S

Analysis of hematological and biochemical parameters revealed several significant differences among groups A, NA, and S (Table 2). Leukocyte counts were highest in group A compared to NA, while group S showed intermediate values. Red blood cell parameters, including erythrocytes, hemoglobin, and hematocrit, were largely similar across groups, with 25% of individuals in each group falling below the lower quartile or above the upper quartile.

Measures of red cell morphology showed significant differences. MCV, MCH, and MCHC varied between groups, with roughly 25% of individuals in group A below or above the quartiles compared to NA and S, indicating subtle changes in red cell size and hemoglobin content. Platelet counts were similar, but RDV-CV and PDW were higher in group A, with 25% of individuals exceeding the upper quartile, reflecting greater heterogeneity in red cell and platelet size.

Eosinophil and basophil counts differed significantly between groups, with group A generally showing lower median values than NA. Neutrophils, lymphocytes, and monocytes were stable, showing similar distributions across groups.

Biochemical markers revealed further differences. Creatine kinase was highest in group S, with 25% of individuals above the upper quartile, whereas group NA had 25% below the lower quartile, indicating pronounced variation in muscle metabolism. Inflammatory markers hs-CRP, SAA, and DII were elevated in group A, with approximately 25% of individuals exceeding the upper quartile, whereas 25% of group S fell below the lower quartile for DII, emphasizing the strongest inflammatory response in group A. Post hoc analyses confirmed significant pairwise differences between groups for these markers.

Overall, while general hematological parameters remained stable, differences in red cell morphology, platelet variability, and inflammatory markers were evident. The quartile analysis highlights that a substantial proportion of group A exhibited elevated inflammatory markers, whereas group S displayed higher muscle enzyme activity, demonstrating distinct physiological and inflammatory profiles among the groups (Table 2).

### 2.3. Principal Component Analysis

Principal component analysis (PCA) was conducted to explore the multivariate relationships between the Dietary Inflammatory Index (DII) and selected inflammatory biomarkers, including high sensitivity C-reactive protein (hs-CRP), serum amyloid A (SAA), total leukocyte count, and leukocyte subpopulations.

#### 2.3.1. Variance Explained by Principal Components

The PCA identified several components describing variability in the dataset. The first three components together explained a substantial proportion of the total variance. The first principal component (PC1) accounted for 24.2% of the variance, the second component (PC2) explained 17.1%, and the third component (PC3) explained 12.8% (Table 3). The first two components together explained approximately 41.3% of the total variance, which is considered acceptable for biological datasets characterized by high physiological variability. Therefore, further interpretation focused primarily on PC1 and PC2.

#### 2.3.2. Interpretation of Principal Components

Analysis of component loadings showed that the first principal component was strongly associated with markers reflecting systemic inflammatory status. The highest loadings were observed for total leukocyte count and neutrophils (0.52 each), followed by SAA (0.45) and hs-CRP (0.41) (Table 4). These variables are well-established indicators of inflammatory activation, suggesting that PC1 represents an axis describing overall systemic inflammation. Higher PC1 scores correspond to individuals with elevated leukocyte counts, increased neutrophil levels, and higher concentrations of hs-CRP and SAA in blood.

In contrast, the second principal component was mainly associated with the distribution of leukocyte subpopulations rather than the magnitude of systemic inflammation. Basophils showed the highest loading for PC2 (0.46), followed by monocytes (0.42) and lymphocytes (0.39) (Table 4). These findings indicate that PC2 reflects variation in immune cell composition and balance within the circulating leukocyte population.

#### 2.3.3. Relationship Between DII and Inflammatory Components

The Dietary Inflammatory Index showed relatively low loadings on the principal components. The loading of DII was −0.06 for PC1 and 0.17 for PC2, indicating a weak relationship between dietary inflammatory potential and the major patterns of variation observed among inflammatory biomarkers in this cohort. These results suggest that, in this dataset, the inflammatory potential of the diet was only weakly associated with circulating inflammatory biomarkers. This finding may reflect the characteristics of the studied population, which consisted of young and generally healthy individuals with relatively low levels of systemic inflammation and limited variability in dietary inflammatory potential.

#### 2.3.4. Differences Between Physical Activity Groups

To explore whether physical activity level influenced the multivariate inflammatory profile, mean PCA scores were calculated for each group: non-active (NA), active (A), and highly active (S) (Table 5). Mean PC1 score was lowest in NA (−0.32), intermediate in A (0.05), and slightly higher in S (0.14); however, differences were minor, suggesting that systemic inflammation was only weakly related to physical activity level. This pattern suggests a small tendency toward higher systemic inflammatory marker values in the S group; however, the differences were minor.

Mean PC2 scores differed slightly between groups, with the NA group showing the highest value (0.33), the A group showing a value close to zero (0.01), and the S group showing a negative value (−0.21). This pattern indicates modest differences in immune cell distribution across physical activity categories.

Overall, the PCA results indicate that inflammatory biomarkers form a distinct systemic inflammation axis characterized mainly by leukocyte counts, neutrophils, hs-CRP, and SAA, while immune cell subtype distribution represents a separate dimension of variability. The Dietary Inflammatory Index showed only a weak association with these multivariate inflammatory patterns. Furthermore, the distribution of PCA scores suggested only minor differences between physical activity groups, indicating that inflammatory biomarker variability was not strongly structured by physical activity level in this cohort.

### 2.4. Multivariable Regression Analyses

Multivariable regression analyses were performed to determine whether the associations between physical activity and inflammatory markers remained significant after adjustment for age and body mass (Table 6).

After adjustment, physical activity was not significantly associated with hs-CRP. Neither moderate (A vs. NA: β = −0.176, *p* = 0.145) nor high physical activity (S vs. NA: β = −0.088, *p* = 0.472) predicted hs-CRP levels. Similarly, age and BMI were not significant predictors. The model explained a very small proportion of variance (R^2^ = 0.028).

For SAA, a similar pattern was observed. Physical activity was not significantly associated with SAA, although a borderline trend was noted for moderate activity (A vs. NA: β = 0.207, *p* = 0.080). High activity (S vs. NA: β = 0.169, *p* = 0.158), age, and BMI were not significant predictors. The explanatory power of the model was low (R^2^ = 0.031).

In contrast, physical activity remained a significant independent predictor of DII after adjustment. Both moderate (A vs. NA: β = −0.756, *p* < 0.001) and high physical activity (S vs. NA: β = −1.482, *p* < 0.001) were associated with lower DII scores, indicating a more anti-inflammatory dietary pattern compared to the low-activity group. Age showed a non-significant trend toward lower DII (β = −0.062, *p* = 0.095), while BMI was not associated with DII. The model explained a substantially higher proportion of variance (R^2^ = 0.212), indicating a stronger relationship between physical activity and dietary inflammatory potential.

Overall, these results indicate that, after accounting for age and BMI, physical activity was not independently associated with circulating inflammatory biomarkers (hs-CRP and SAA), but remained strongly associated with dietary inflammatory potential.

## 3. Discussion

The present study investigated the interplay between physical activity, dietary inflammatory potential, and systemic inflammatory markers in healthy young adult men, revealing nuanced associations that underscore the complexity of lifestyle-inflammation relationships. Our findings suggest that anthropometric and body composition measures varied significantly across physical activity levels, with the low-activity group (NA) exhibiting higher adiposity, central fat distribution, and lower fat-free mass compared to both moderately (A) and highly active (S) participants. These results are consistent with previous evidence that regular physical activity, particularly at moderate to high levels, may support favorable body composition by reducing fat mass and increasing lean tissue, thereby contributing to enhanced metabolic efficiency and basal metabolic rate [10].

Hematological analysis revealed subtle but significant differences in red cell morphology and platelet indices among the groups, while general leukocyte counts remained largely stable. Interestingly, the moderate activity group (A) exhibited higher median leukocyte counts, slightly elevated CRP and SAA levels, and increased variability in red cell distribution width (RDW-CV) and platelet distribution width (PDW). These findings may suggest that moderate but structured physical activity could transiently stimulate mild inflammatory and hematological responses, potentially reflecting adaptive physiological stress rather than pathological inflammation [11]. In contrast, highly active individuals (S) displayed elevated creatine kinase levels, consistent with increased skeletal muscle turnover and metabolic demands associated with intensive training, without a concomitant rise in systemic inflammatory markers, which may suggest efficient recovery mechanisms and adaptation to regular high-intensity exercise [12].

Principal component analysis further elucidated the multivariate structure of inflammatory biomarkers, revealing two major axes: PC1 representing systemic inflammatory activity (leukocytes, neutrophils, CRP, and SAA) and PC2 reflecting immune cell distribution (basophils, monocytes, and lymphocytes). Notably, the Dietary Inflammatory Index demonstrated weak associations with both PCs, which may indicate that dietary inflammatory potential had a limited influence on circulating inflammatory biomarkers in this young and generally healthy cohort. This observation is in line with prior studies suggesting that the relationship between diet-induced inflammation and circulating markers may be more pronounced in older, metabolically compromised, or clinically at-risk populations [13,14]. Shivappa et al. [13] demonstrated an association between CRP and the DII ratio in healthy middle-aged men, although this applied only to CRP values above 3 mg/L. It cannot be ruled out that, in a population of young, healthy men, the association between DII and CRP only emerges when the diet becomes distinctly pro-inflammatory, rather than moderately pro-inflammatory or anti-inflammatory, as was the case with the groups of men studied. However, further observations would be needed to confirm this. The omission of certain bioactive compounds, such as flavonoids and other micronutrients, may have contributed to the relatively weak associations observed in our study. These compounds are known to exert various biological effects, including antioxidant and anti-inflammatory activities [15]. Although this did not prevent comparisons between groups, it may have influenced the relationship between the DII and the inflammatory markers under investigation.

Despite minor differences in PCA scores across physical activity groups, the data suggest that systemic inflammatory variability was not strongly structured by habitual activity in this population. While the moderate activity group exhibited slightly higher systemic inflammation (PC1) than the low-activity group, the highly active participants did not display marked elevations, which may support the notion that exercise intensity, frequency, and adaptation modulate inflammatory responses in a non-linear manner [16]. These findings are consistent with the emerging view that physical activity modulates inflammation in a dose-dependent and context-specific fashion, whereby moderate activity may transiently elevate immune parameters, while chronic high-level training may either maintain or suppress low-grade systemic inflammation through adaptation mechanisms [17].

Importantly, multivariable regression analyses indicated that the associations between physical activity and circulating inflammatory markers (hs-CRP and SAA) were no longer significant after adjustment for age and BMI. This may suggest that the differences observed in unadjusted analyses could be partially explained by variation in body composition or age across groups.

In contrast, physical activity remained a significant independent predictor of the DII, with higher activity levels associated with more anti-inflammatory dietary patterns. This finding may indicate a clustering of health-related behaviors, whereby physically active individuals are more likely to follow diets with lower inflammatory potential.

Collectively, the study highlights the differential physiological and inflammatory profiles across activity levels and underscores the importance of considering both body composition and exercise adaptation when interpreting biomarkers of systemic inflammation. The present findings suggest that, in young healthy men, circulating inflammatory markers may be relatively insensitive to differences in habitual physical activity once key confounders are taken into account. The weak association between DII and inflammation may indicate that in healthy young men with limited dietary variability, other factors, such as exercise-induced muscle stress, circadian rhythm, and innate immune responsiveness, could exert a more substantial influence on circulating inflammatory markers [18].

## 4. Material and Methods

### 4.1. Characteristics of the Study Participants

This study was designed as a cross-sectional observational investigation examining associations between physical activity level, dietary inflammatory potential, and systemic inflammatory markers in healthy young adult men [19]. The study was reported in accordance with the Strengthening the Reporting of Observational Studies in Epidemiology (STROBE) guidelines for cross-sectional studies. The study included young men aged 18–30 years. Inclusion criteria were: age ≥ 18 and ≤30 years, good health status (no chronic diseases or injuries), no use of medications, non-smoking status, and written informed consent to participate in the study. Exclusion criteria included performing physical labor and the occurrence of illness or injury during the study period.

Participants were recruited through university announcements. Eligibility screening was performed prior to enrollment by study investigators.

A total of 250 men initially agreed to participate; however, the final analysis included 233 participants, as data from 17 individuals were excluded due to incomplete participation in all stages of the study. Participants with incomplete anthropometric, dietary, or biochemical data were excluded using complete-case analysis.

Participants were classified into three groups according to their level of physical activity, representing low, moderate, and high levels of activity. Physical activity groups were predefined based on structured weekly training volume and curricular sports participation to reflect low, moderate, and high habitual activity categories.

The first group (A) consisted of students of physical education or sport at the Józef Piłsudski University of Physical Education in Warsaw, whose physical activity resulted solely from participation in sports classes included in the study curriculum (*n* = 93). Their weekly physical activity ranged from 3 to 7 h, representing a moderate level of physical activity. Energy expenditure on physical activity in this group was 2190 ± 301 kcal/d. The mean age of participants in this group was 21 years.

The second group (S) consisted of students from the same university who participated both in mandatory sports classes and additional structured sports training (*n* = 88). These participants trained in strength, strength–speed, and strength–endurance disciplines, including weightlifting, martial arts (karate, kickboxing, judo), artistic gymnastics, bodybuilding (classic bodybuilding and men’s physique), and tennis. All athletes were in the preparatory phase of their training macrocycle, and their weekly physical activity exceeded 7 h, representing a high level of physical activity. Energy expenditure in this group amounted to 3149 ± 669 kcal/d. The mean age of this group was 25 years.

The control group (NA) consisted of students from the Warsaw University of Life Sciences or the Warsaw University of Technology whose study programs did not include courses requiring increased physical effort and who did not engage in regular additional physical activity (*n* = 52). This group was characterized by a low level of physical activity, with a mean daily energy expenditure of 485 ± 210 kcal. The mean age of the control group was 23 years.

Thus, the study population represented three distinct levels of physical activity: low (NA), moderate (A), and high (S). The sample size reflected a convenience sample of eligible volunteers available during the recruitment period. No formal a priori power calculation was performed.

### 4.2. Study Procedures

**Figure 1:**
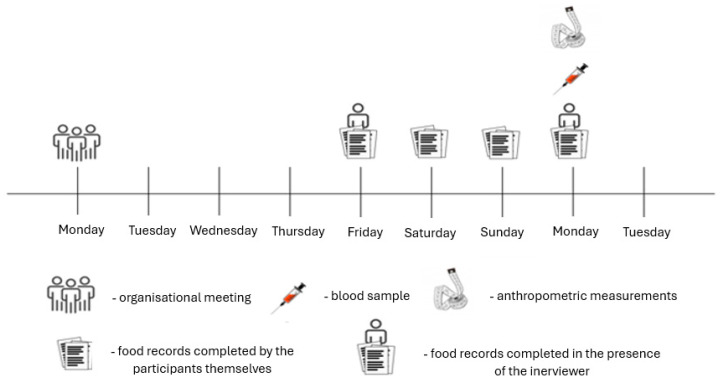
Study timeline.

Recruitment and data collection were conducted in Warsaw, Poland, at the Józef Piłsudski University of Physical Education. Blood sampling and anthropometric measurements were performed every Monday. One week before the study, an informational meeting was held to explain the study procedures, objectives, and participants’ right to withdraw at any stage. The study timeline is presented in Figure 1. To minimize measurement bias, all anthropometric and blood sampling procedures were performed by trained personnel using standardized protocols. Participants received uniform written and verbal instructions regarding pre-assessment preparation. Laboratory analyses were conducted in a certified diagnostic laboratory blinded to participants’ physical activity classification.

All procedures were performed in accordance with the Declaration of Helsinki, and the research protocol received ethical approval from the Senate Committee for Research Ethics at the Józef Piłsudski University of Physical Education in Warsaw (SKE 01-18/2017).

### 4.3. Anthropometric Measurements

Body weight and height were measured using standardized procedures and calibrated equipment. Body composition, including body fat percentage, was assessed using tetrapolar bioelectrical impedance analysis (BIA) with the BC-418 device (Tanita Co., Tokyo, Japan). To evaluate fat distribution, the waist-to-hip ratio (WHR) and waist-to-height ratio (WHtR) were calculated. Waist and hip circumferences were measured with a flexible tape measure, with a measurement accuracy of 0.5 cm.

Participants were instructed to follow standard pre-test procedures in order to minimise, in particular, variations in bioelectrical impedance measurements. They were required to refrain from consuming caloric food and drink for at least 8 h prior to the test, to avoid strenuous physical activity on the day prior to the test, to refrain from consuming alcohol for at least 24 h before the assessment, to maintain normal hydration whilst avoiding excessive fluid intake within 2 h of the measurement, and to empty their bladder within 30 min of the test. All measurements were taken in the morning, between 8:00 and 10:00, with participants wearing sportswear and walking barefoot.

### 4.4. Dietary Assessment and Dietary Inflammatory Index (DII)

Dietary intake was assessed using 4-day food records completed by the participants, including information on all foods, beverages, and dietary supplements consumed. Two of the days were weekdays, and two were weekend days. Weekday records were completed under the supervision of a trained researcher, while weekend records were self-reported by participants after receiving detailed instructions on proper record-keeping. Portion sizes were identified using the Food and Dish Photo Album developed by the Institute of Food and Nutrition, Warsaw. Nutrient and energy intake were calculated using Dieta 5.0 software (National Institute of Public Health—National Institute of Hygiene, Warsaw, Poland), also developed by the Institute of Food and Nutrition in Warsaw.

The Dietary Inflammatory Index (DII) was calculated according to the method of Shivappa et al. [20]. Individual intakes of each food component were standardized against global mean intakes (Z-scores), converted to percentile scores (PS), and then centered (centered percentile value, CPV) to a symmetrical distribution around 0, ranging from −1 (maximally anti-inflammatory) to +1 (maximally pro-inflammatory). Each CPV was multiplied by the corresponding overall inflammatory effect score for that nutrient, yielding the DII for individual dietary components. The total DII for each participant was obtained by summing the component-specific DII values.

In this study, DII was calculated using 37 of the 45 original components, as some nutrients, including flavan-3-ols, flavones, flavonols, flavanones, anthocyanidins, trans fats, and selenium, could not be reliably assessed using the Dieta 5.0 software. The food parameters taken into account were: energy, carbohydrate, protein, total fat, alcohol, fiber, cholesterol, saturated fat, MUFA, PUFA, omega-3, omega-6, niacin, thiamin, riboflavin, vitamin B12, B6, iron, magnesium, zinc, vitamin A, β-carotene, vitamin C, vitamin D, vitamin E, folic acid, caffeine, garlic, ginger, onion, pepper, rosemary, saffron, oregano, turmeric, eugenol and tea (green, black).

### 4.5. Biochemical Analyses

Blood samples were collected in the Laboratory of Human Biology at the Józef Piłsudski University of Physical Education in Warsaw by a trained phlebotomist. Sampling was performed in the morning from the antecubital vein, after an overnight fast, and participants refrained from physical activity the day before blood collection.

Creatine kinase (CK), high-sensitivity C-reactive protein (hs-CRP), and complete blood count with smear were analyzed on the day of collection in a certified laboratory (Diagnostyka sp. z o.o., Warsaw, Poland). Plasma for serum amyloid A (SAA) measurement was obtained by centrifuging blood for 10 min at 4000 rpm and stored at −70 °C until analysis. Blood was collected into Primavette V vacuum tubes (KABE Labortechnik, Nümbrecht, Germany). Complete blood count with smear was performed using flow cytometry, calorimetry, and optical microscopy. Reference ranges for hematological parameters were: leukocytes 4.2–9.1 × 10^3^/µL, erythrocytes 4.6–6.1 × 10^6^/µL, hemoglobin 13.7–17.5 g/dL, hematocrit 40–51%, MCV 79–92 fL, MCH 26–32 pg, MCHC 32.3–36.5 g/dL, platelets 150–400 × 10^3^/µL, RDV-CV 11.6–14.4%, PDW 9.8–16.1%, MPV 9.4–12.6 fL, P-LCR 19.2–47%, neutrophils 2.0–7.0 × 10^3^/µL (40–80%), lymphocytes 1.0–3.0 × 10^3^/µL (20–40%), monocytes 0.02–1 × 10^3^/µL (2–10%), eosinophils 0.02–0.5 × 10^3^/µL (1–6%), basophils 0.02–0.1 × 10^3^/µL (0–2%).

CK activity was measured by spectrophotometry with a sensitivity of 5 U/L; reference values for men range from 30 to 200 U/L. Hs-CRP concentration was determined using an immunoturbidimetric assay with a sensitivity of 0.3 mg/L, and reference values are <5 mg/L. SAA concentration was measured using an enzyme-linked immunosorbent assay (ELISA) (Invitrogen™ Human SAA ELISA Kit, Thermo Fisher Scientific, Waltham, MA, USA) with a sensitivity of <5 pg/mL, and reference values are <10 mg/L.

### 4.6. Statistical Analyses

Statistical analyses were performed using PQStat v. 1.8.4 (PQStat Software, Poznań, Poland) and Matlab R2021a (MathWorks Inc., Natick, MA, USA). The level of statistical significance was set at *p* < 0.05.

The distribution of all variables was assessed using the Shapiro–Wilk test. As the majority of variables showed non-normal distributions, non-parametric statistical methods were applied. Descriptive statistics are presented as median and interquartile range (IQR).

Differences between the three physical activity groups (NA—low, A—moderate, S—high) were assessed using the Kruskal–Wallis test. When significant differences were detected, Dunn’s post hoc test with Bonferroni correction was applied for pairwise comparisons. To evaluate the magnitude of between-group differences, effect size (*ε*^2^) for the Kruskal–Wallis test was calculated according to the formula:

$$\varepsilon^{2}=\frac{H-k+1}{n-k}$$

where *H* is the Kruskal–Wallis test statistic, *k* is the number of groups, and *n* is the total sample size. Effect sizes were interpreted as small (>0.01), medium (>0.08), and large (>0.26) [21].

To explore multivariate relationships between the Dietary Inflammatory Index (DII) and selected inflammatory biomarkers, principal component analysis (PCA) was performed. The analysis included the following variables: C-reactive protein (CRP), serum amyloid A (SAA), total leukocyte count, and leukocyte subpopulations (neutrophils, lymphocytes, monocytes, eosinophils, and basophils).

Prior to PCA, all variables were standardized using a Z-score transformation to eliminate the influence of different measurement scales. Principal components were extracted based on eigenvalues greater than 1 and inspection of the scree plot. The proportion of total variance explained by each component was calculated, and factor loadings were examined to determine the contribution of individual variables to each principal component.

Additionally, multivariable linear regression analyses were performed to assess whether the associations between physical activity level and inflammatory markers (hs-CRP, SAA) as well as the Dietary Inflammatory Index (DII) remained independent of potential confounders. Physical activity group (NA, A, S) was included as a categorical variable (reference: NA—low activity), while age and body mass index (BMI) were entered as covariates. Due to non-normal distributions, hs-CRP and SAA were log-transformed prior to analysis. Regression results are presented as standardized regression coefficients (β), 95% confidence intervals (CI), and *p*-values. Model fit was evaluated using the coefficient of determination (R^2^) and adjusted R^2^.

## 5. Conclusions

This study provides insight into the complex interactions between physical activity, dietary patterns, and systemic inflammation in healthy young men. Moderate physical activity was associated with lower adiposity but slightly elevated inflammatory markers, whereas high physical activity corresponded to increased muscle turnover without a substantial inflammatory burden. Dietary inflammatory potential showed minimal influence on circulating biomarkers in this cohort. While these findings may suggest that exercise-related physiological stress could play a role in shaping inflammatory profiles among young adults, this observation should be interpreted with caution given the cross-sectional design and relatively weak associations. This should be considered a plausible hypothesis that warrants further investigation. Overall, these findings emphasize the importance of considering both activity level and body composition in evaluating systemic inflammation and support the notion that regular, well-adapted physical activity promotes favorable health outcomes while maintaining controlled inflammatory responses. Future studies incorporating longitudinal designs, diverse populations, and comprehensive biomarker panels are needed to further clarify the synergistic effects of diet and exercise on immune regulation.

### Study Limitation

Several limitations warrant consideration. First, the cross-sectional design precludes causal inferences between physical activity, diet, and inflammatory status. Longitudinal studies are required to disentangle temporal relationships and adaptation effects. Second, dietary assessment relied on self-reported 4-day food records, which are susceptible to recall bias and underreporting, particularly for nutrients contributing to DII calculations such as polyphenols and flavonoids. Moreover, the DII was calculated based on an incomplete set of the original 45 components, which may have influenced the accuracy and comparability of the inflammatory potential estimates. Third, the study population consisted exclusively of young, healthy men, limiting generalizability to women, older adults, or clinical populations with higher inflammatory burden. Selection bias cannot be excluded because participants were recruited from university populations and may not be representative of the general young adult male population. Additionally, age differences between the analyzed groups may have acted as a confounding factor influencing both dietary patterns and inflammatory markers. The sporting heterogeneity within group S should also be acknowledged, as participants represented different types of physical activity, which may vary in their physiological and inflammatory effects. Fourth, although multivariable regression models were applied to adjust for key confounders such as age and BMI, residual confounding from unmeasured factors (e.g., sleep, stress, or unrecorded dietary components) cannot be excluded. Fifth, while BIA provides practical estimates of body composition, it is less precise than gold-standard methods such as dual-energy X-ray absorptiometry (DXA). Finally, only selected inflammatory biomarkers were assessed; inclusion of additional cytokines (e.g., IL-6, TNF-α) and markers of oxidative stress could provide a more comprehensive picture of systemic inflammation. Therefore, extrapolation of these findings to women, non-student populations, older adults, or individuals with chronic disease should be undertaken cautiously.

## Figures and Tables

**Table 1:** Anthropometric and body composition characteristics of the study groups (NA, A, S). Data are presented as median (IQR). Differences were assessed using the Kruskal–Wallis test followed by Dunn–Bonferroni post hoc test. Different superscript letters (^a–c^) indicate significant differences between groups (*p* < 0.05).

Variable	NA (Low)	A (Moderate)	S (High)	H (2)	*p*-Value	$\varepsilon^{2}$
Age [years]	24 (22–25) ^a^	21 (20–23) ^b^	25 (23–27) ^c^	71.92	<0.001	0.24
Body mass [kg]	84.3 (76.6–96.1) ^a^	78 (72.8–82) ^b^	85.3 (79.9–90.1) ^a^	41.44	<0.001	0.12
Body height [cm]	179 (176.8–181)	180 (177–184)	180 (177.7–184.2)	3.14	0.211	0.01
BMI [kg/m^2^]	26.7 (24.2–30.0) ^a^	23.9 (22.3–24.9) ^b^	26.1 (24.9–27.6) ^a^	53.98	<0.001	0.18
BMR [kcal]	1969.5 (1841.75–2228) ^a^	1963 (1880–2040) ^a^	2113 (1980.75–2275) ^b^	28.75	<0.001	0.11
Fat [%]	23.3 (20.9–26.3) ^a^	11.9 (9.4–13.7) ^b^	12.4 (10.68–14.57) ^b^	116.51	<0.001	0.34
Fat mass [kg]	19.9 (17.77–23.62) ^a^	8.9 (7.3–11) ^b^	10.55 (8.85–12.97) ^b^	120.77	<0.001	0.36
FFM [kg]	63 (57.675–76) ^a^	68.7 (65.1–71.7) ^b^	74.5 (70–78.75) ^c^	43.1	<0.001	0.13
WHtR	0.50 (0.47–0.52) ^a^	0.44 (0.41–0.45) ^b^	0.47 (0.44–0.49) ^c^	81.05	<0.001	0.28
WHR	0.91 (0.88–0.92) ^a^	0.81 (0.79–0.83) ^b^	0.88 (0.85–0.90) ^c^	116.71	<0.001	0.34

BMI—body mass index; BMR—basal metabolic rate; FFM—fat-free mass; WHtR—waist-to-height ratio; WHR—waist-to-hip ratio.

**Table 2:** Hematological and biochemical parameters across study groups. Data are presented as median (IQR). Differences were assessed using the Kruskal–Wallis test followed by Dunn–Bonferroni post hoc test. Different superscript letters (^a–c^) indicate significant differences between groups (*p* < 0.05).

Variable	NA	A	S	H (2)	*p*-Value	$\varepsilon^{2}$
Hematological parameters						
Leukocytes [×10^3^/µL]	6.05 (5.9–6.43) ^a^	6.6 (5.7–7.4) ^b^	6.3 (5.3–7.02) ^ab^	7.78	0.021	0.03
Erythrocytes [×10^6^/µL]	5.1 (4.9–5.3)	5.2 (4.9–5.4)	5.2 (5–5.5)	5.63	0.062	0.03
Hemoglobin [g/dL]	15.4 (15–16.1)	15.3 (14.9–16.1)	15.45 (15.1–16)	1.29	0.521	-
Hematocrit [%]	45 (44–46.25)	45 (44–46)	45 (44–46.25)	0.19	0.910	-
Red blood cell indices						
MCV [fL]	88 (87–89.25) ^a^	87 (85–89) ^b^	88 (86–89) ^ab^	7.06	0.032	0.03
MCH [pg]	30 (29.75–31) ^a^	30 (29–31) ^ab^	30 (29–30) ^b^	12.44	0.010	0.05
MCHC [g/dL]	34 (33.175–35.1) ^a^	34.7 (33.8–35.5) ^b^	34.2 (33.5–34.9) ^ab^	7.39	0.020	0.05
RDV-CV [%]	12.7 (12.4–13) ^a^	12.9 (12.3–13.3) ^ab^	13.2 (12.675–13.7) ^b^	17.63	0.010	0.05
Platelet parameters						
Platelets [×10^3^/µL]	208.5 (183–260.5)	222 (198–266)	231.5 (198.75–258.75)	2.54	0.280	-
PDW [%]	13.8 (12.9–14.35) ^a^	14.3 (13.2–15) ^b^	13.5 (12.7–14.35) ^a^	8.57	0.010	0.03
MPV [fL]	11.05 (10.4–12.32)	11.2 (10.6–11.8)	11 (10.6–12.05)	0.25	0.880	-
P-LCR [%]	35.1 (32.27–37.57)	34.8 (28.5–39.2)	34.05 (29.32–38.6)	1.00	0.600	-
Leukocyte subpopulations (absolute)						
Neutrophils [×10^3^/µL]	3.21 (2.79–3.59)	3.2 (2.58–3.82)	3.39 (2.7–4.00)	2.03	0.360	-
Lymphocytes [×10^3^/µL]	2.1 (1.8–2.4)	2.3 (1.9–2.7)	2.1 (2–2.5)	3.51	0.170	-
Monocytes [×10^3^/µL]	0.62 (0.53–0.74)	0.61 (0.49–0.69)	0.58 (0.49–0.70)	2.64	0.260	0.09
Eosinophils [×10^3^/µL]	0.2 (0.13–0.25) ^a^	0.15 (0.09–0.22) ^b^	0.185 (0.12–0.2625) ^ab^	8.23	0.020	0.05
Basophils [×10^3^/µL]	0.03 (0.02–0.04) ^a^	0.02 (0.01–0.03) ^b^	0.02 (0.02–0.03) ^b^	7.98	0.020	0.09
Leukocyte subpopulations [%]						
Neutrophils [%]	54.25 (48.375–59.5)	52 (49.1–58.4)	51.2 (48.3–56.225)	2.23	0.330	-
Lymphocytes [%]	35.7 (29.575–39.2)	34.4 (29.1–38.4)	36.45 (30.8–39.55)	3.33	0.190	-
Monocytes [%]	8.85 (7.6–9.55) ^a^	9.6 (8.6–10.6) ^b^	8.35 (7.375–9.4) ^a^	25.54	0.010	0.09
Eosinophils [%]	3.45 (2–4.3) ^a^	2.2 (1.2–3.1) ^b^	2.75 (2–3.4) ^b^	24.56	0.010	0.09
Basophils [%]	0.45 (0.3–0.525)	0.4 (0.3–0.6)	0.4 (0.3–0.5)	2.03	0.360	-
Biochemical parameters						
Creatine kinase [U/L]	95 (72.75–118.5) ^a^	228 (160–289) ^b^	369 (216–469.75) ^c^	101.74	<0.010	0.32
hs-CRP [mg/L]	0.9 (0.67–1.4) ^ab^	0.6 (0.3–1.6) ^a^	0.9 (0.5–1.7) ^b^	7.92	0.020	0.03
SAA [mg/L]	1.41 (1.02–2.36) ^a^	2.4 (1.6–3.5) ^b^	2.5 (1.45–3.69) ^b^	17.48	0.010	0.05
DII [index]	1.53 (0.48–2.57) ^a^	0.73 (−0.71–1.61) ^b^	−0.68 (−1.52–0.01) ^c^	72.31	<0.010	0.29

**Table 3:** Variance explained by the first three principal components.

Principal Component	PC1	PC2	PC3
Variance Explained [%]	24.2	17.1	12.8

**Table 4:** Major loadings contributing to PC1 and PC2.

Variable	Leukocytes	Neutrophils	SAA	hs-CRP	Basophils	Monocytes	Lymphocytes
PC1 loading	0.52	0.52	0.45	0.41	0.20	0.25	0.18
PC2 loading	0.15	0.08	0.12	0.10	0.46	0.42	0.39

**Table 5:** Mean PCA scores according to physical activity group.

Group	PC1	PC2
NA	−0.32	0.33
A	0.05	0.01
S	0.14	−0.21

**Table 6:** Multivariable regression models for hs-CRP, SAA, and DII adjusted for age and BMI.

Outcome Variable	Predictor	β Coefficient	95% CI	*p*-Value
hs-CRP (log)	Physical activity (A vs. NA)	−0.176	−0.412 to 0.060	0.145
	Physical activity (S vs. NA)	−0.088	−0.329 to 0.153	0.472
	Age	0.019	−0.014 to 0.052	0.258
	BMI	0.011	−0.003 to 0.025	0.121
	Model R^2^/adj. R^2^	0.028/0.011		
SAA (log)	Physical activity (A vs. NA)	0.207	−0.025 to 0.439	0.080
	Physical activity (S vs. NA)	0.169	−0.066 to 0.404	0.158
	Age	0.015	−0.017 to 0.047	0.356
	BMI	0.009	−0.004 to 0.022	0.174
	Model R^2^/adj. R^2^	0.031/0.014		
DII	Physical activity (A vs. NA)	−0.756	−1.158 to −0.354	<0.001 *
	Physical activity (S vs. NA)	−1.482	−1.912 to −1.052	<0.001 *
	Age	−0.062	−0.135 to 0.011	0.095
	BMI	0.018	−0.004 to 0.040	0.112
	Model R^2^/adj. R^2^	0.212/0.198		

Physical activity group was included as a categorical variable (reference: NA—low activity). hs-CRP and SAA were log-transformed prior to analysis due to non-normal distributions. BMI—body mass index. β—standardized regression coefficient; CI—confidence interval; R^2^—coefficient of determination; adj. R^2^—adjusted R^2^. Statistically significant values (*p* < 0.05) are denoted by asterisk (*).

## Data Availability

The dataset supporting the findings of this study is publicly available in the Zenodo repository at https://doi.org/10.5281/zenodo.19056738 under the Creative Commons Attribution 4.0 International (CC BY 4.0) license.
